# Optic disc edema and chorioretinal folds develop during strict 6° head‐down tilt bed rest with or without artificial gravity

**DOI:** 10.14814/phy2.14977

**Published:** 2021-08-06

**Authors:** Steven S. Laurie, Scott H. Greenwald, Karina Marshall‐Goebel, Laura P. Pardon, Akash Gupta, Stuart M. C. Lee, Claudia Stern, Haleh Sangi‐Haghpeykar, Brandon R. Macias, Eric M. Bershad

**Affiliations:** ^1^ KBR Houston TX USA; ^2^ Center for Space Medicine Baylor College of Medicine Houston TX USA; ^3^ Institute of Aerospace Medicine German Aerospace Center Clinical Aerospace Medicine Cologne Germany; ^4^ Department of Obstetrics and Gynecology Baylor College of Medicine Houston TX USA; ^5^ NASA Johnson Space Center Houston TX USA; ^6^ Department of Neurology Baylor College of Medicine Houston TX USA; ^7^ Department of Neurosurgery Baylor College of Medicine Houston TX USA

**Keywords:** artificial gravity, bed rest, centrifugation, chorioretinal folds, retinal thickness, spaceflight analog, spaceflight associated neuro‐ocular syndrome

## Abstract

Spaceflight associated neuro‐ocular syndrome (SANS) is hypothesized to develop as a consequence of the chronic headward fluid shift that occurs in sustained weightlessness. We exposed healthy subjects (*n* = 24) to strict 6° head‐down tilt bed rest (HDTBR), an analog of weightlessness that generates a sustained headward fluid shift, and we monitored for ocular changes similar to findings that develop in SANS. Two‐thirds of the subjects received a daily 30‐min exposure to artificial gravity (AG, 1 g at center of mass, ~0.3 g at eye level) during HDTBR by either continuous (cAG, *n* = 8) or intermittent (iAG, *n* = 8) short‐arm centrifugation to investigate whether this intervention would attenuate headward fluid shift‐induced ocular changes. Optical coherence tomography images were acquired to quantify changes in peripapillary total retinal thickness (TRT), retinal nerve fiber layer thickness, and choroidal thickness, and to detect chorioretinal folds. Intraocular pressure (IOP), optical biometry, and standard automated perimetry data were collected. TRT increased by 35.9 µm (95% CI, 19.9–51.9 µm, *p* < 0.0001), 36.5 µm (95% CI, 4.7–68.2 µm, *p* = 0.01), and 27.6 µm (95% CI, 8.8–46.3 µm, *p* = 0.0005) at HDTBR day 58 in the control, cAG, and iAG groups, respectively. Chorioretinal folds developed in six subjects across the groups, despite small increases in IOP. Visual function outcomes did not change. These findings validate strict HDTBR without elevated ambient CO_2_ as a model for investigating SANS and suggest that a fluid shift reversal of longer duration and/or greater magnitude at the eye may be required to prevent or mitigate SANS.

## Graphical abstract

Spaceflight associated neuro‐ocular syndrome (SANS) is hypothesized to develop as a consequence of the chronic headward fluid shift that occurs in sustained weightlessness. To investigate this condition, healthy subjects were exposed to strict 6º head‐down tilt bed rest (HDTBR), a spaceflight analog that causes a chronic headward fluid shift and can lead to the development of optic disc edema in some participants. For the first time, we demonstrate that chorioretinal folds develop during HDTBR, along with signs of optic disc edema, similar to findings that occur in astronauts during spaceflight. However, ocular changes were not mitigated in subjects who were exposed for 30 min per day to continuous or intermittent artificial gravity (AG) via centrifugation during HDTBR. These findings validate strict HDTBR without elevated ambient CO_2_ as a model for investigating these ocular changes and suggest that a fluid shift reversal of longer duration and/or greater magnitude at the eye than used here may be required to prevent or mitigate spaceflight associated neuro‐ocular syndrome findings.

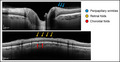

## 1 INTRODUCTION

In the sentinel case series describing the condition that is now known as spaceflight associated neuro‐ocular syndrome (SANS), fundoscopic examination of seven astronauts who had returned from long‐duration missions to the International Space Station (ISS) showed that six of the astronauts had optic disc edema or chorioretinal folds (Mader et al., 2011). More recently, both features have been observed during spaceflight using optical coherence tomography (OCT), which provides a more sensitive assessment of these changes than fundoscopy (Laurie, Lee, et al., 2019; Macias et al., 2020; Mader et al., 2017). Identified by increased peripapillary total retinal thickness (TRT), optic disc edema has been observed as early as the tenth day of spaceflight, with progressive thickening and radial expansion occurring over the course of the missions and recovery to baseline within 3 months of return to Earth (Laurie, Lee, et al., 2019; Macias et al., 2020). In some individuals, chorioretinal folds have persisted years after return to Earth (Lee et al., 2020; Mader et al., 2011). Although SANS is widely hypothesized to result from a chronic headward fluid shift induced by weightlessness (Zhang & Hargens, 2017), the underlying mechanisms are not well understood. Therefore, countermeasure development has been primarily focused on strategies that reverse the headward fluid shift.

We recently reported that optic disc edema develops in healthy subjects exposed to 30 days of strict 6˚ head‐down tilt bed rest (HDTBR) in a 0.5% CO_2_ atmosphere (Laurie, Lee, et al., 2019; Laurie, Macias, et al., 2019). Despite finding no change in arterialized or end‐tidal partial pressure of CO_2_ levels in those subjects (Laurie et al., 2020; Laurie, Macias, et al., 2019), it remained unclear if it was the mild hypercapnic environment or the strict HDTBR that caused the development of optic disc edema because this was the first time that either strict head‐down tilt or elevated ambient CO_2_ had been implemented in this type of experiment. Here, we tested whether optic disc edema can be induced by strict 6˚ HDTBR in a normocapnic environment by studying subjects participating in the *Artificial Gravity Bed Rest with the European Space Agency (AGBRESA)* bed rest campaign. Furthermore, a daily 30‐min exposure to continuous or intermittent artificial gravity (AG) by short‐arm centrifugation was applied to a subset of the subjects during HDTBR, as predefined in the AGBRESA protocol, to test whether this countermeasure can prevent the development of optic disc edema and other SANS findings.

## 2 METHODS

### 2.1 Ethical approval

Twenty‐four subjects participated in this study, which was conducted at the :envihab facility at the German Aerospace Center (DLR) in Cologne, Germany (Frett et al., 2020). Subjects were recruited by the DLR and eligibility for enrollment was determined according to the International Academy of Astronautics’ guidelines for bed rest studies (Sundblad et al., 2014), including a clinical eye examination that confirmed the absence of ocular disease. Informed consent was obtained from the subjects in writing after explanation of the nature of the study and possible consequences. Data were collected between March and December of 2019 in accordance with the study protocol approved by the NASA Johnson Space Center Institutional Review Board and the Medical Association of North Rhine (Ärztekammer Nordrhein). This study adhered to the Declaration of Helsinki and The Physiological Society's policies regarding human experiments, and it is included in the German Clinical Trials Register (DRKS00015677).

### 2.2 Environment

Each subject lived at the :envihab facility in Cologne, Germany for 89 days. Baseline data collection (BDC) occurred across the initial 14 days (BDC‐14 through BDC‐1), followed by 60 days of strict 6° head‐down tilt bed rest (HDT1 through HDT60), and then recovery (R) data collection across the final 14 days (R+0 through R+13). Subjects were ambulatory during the BDC and recovery periods, although they were not permitted to leave the facility. As previously described (Marshall‐Goebel et al., 2017; Laurie, Macias, et al., 2019; Laurie, Lee, et al., 2019; Lee, et al., 2020), no pillow was allowed during the bed rest phase of the study, except for an approved contoured cushion for side sleeping, and subjects were required to have a least one shoulder in contact with the mattress at all times (which was monitored) to ensure that a full‐body 6˚ HDT angle was maintained. Individual nutritional requirements were calculated from the basal metabolic measurements on BDC‐14. Dietary intake was monitored by the :envihab staff, and the meals adhered to the NASA and European Space Agency standardization plan, as previously described (Laurie et al., 2020).

### 2.3 Study design

The study design, including the number of subjects and AG centrifugation profiles, was predefined in the NASA Human Exploration Research Opportunities NNJ15ZSA001N‐AGBR, Appendix G. The cohort was divided by semi‐random assignment into three groups (*n* = 8 per group) that were balanced for sex, age, height, and weight distributions (Table 1). Subjects in the control group received no intervention. The remaining two groups were exposed daily to 30 min of either continuous AG (cAG) or intermittent AG (iAG) by short‐arm centrifugation during the HDTBR phase. The iAG exposure consisted of 6 periods of AG that lasted 5 min each, with a 3‐min break between each period. The centrifuge arm radius and velocity were adjusted according to each subject's height to achieve 1 g at the center of mass (approximately 2 g at the feet and 0.3 g at the eyes) (Frett et al., 2020). All centrifugation sessions were performed in the 0° supine posture, and on testing days, ocular measures were acquired from the cAG and iAG subjects before the centrifugation session. During data analysis, the study team was masked to each subject's experimental group assignment.

**TABLE 1:** Subject demographics and morphometrics

	Control	cAG	iAG	Total
Cohort size	8 (6 male/2 female)	8 (5 male/3 female)	8 (5 male/3 female)	24 (16 male/8 female)
Age (years)	34 ± 8	32 ± 10	34 ± 11	33 ± 9
Height (cm)	177 ± 7	173 ± 8	174 ± 11	175 ± 9
Weight (kg)	79 ± 13	72 ± 10	71 ± 5	74 ± 10
BMI (kg/m^2^)	25 ± 3	24 ± 2	24 ± 2	24 ± 2

Cohort sizes and characteristics. Values represent mean ± SD.

Abbreviations: BMI, body mass index; cAG, continuous artificial gravity; iAG, intermittent artificial gravity.

### 2.4 Optical coherence tomography

Optical coherence tomography scans of the optic nerve head (ONH) and adjacent retina were acquired using the Spectralis OCT2 camera mounted on the Flex Module (Heidelberg Engineering) on BDC‐6, HDT2, HDT15, HDT31, HDT45, HDT58, R+6, and R+11 to evaluate changes in ocular structure. All OCT images were acquired using the AutoRescan feature to ensure accurate alignment of follow‐up scans for individual subjects. Images were acquired in the seated and supine postures before HDTBR, in the 6º HDT posture during HDTBR, and in the supine posture after HDTBR. For pre‐ and post‐HDTBR sessions, subjects acclimated to each posture for at least 5 min before data were collected for that posture.

A 24‐line, 20º radial scan pattern centered on the ONH was used to quantify changes in TRT. Two independent readers manually corrected automated internal limiting membrane (ILM) and Bruch's membrane (BM) segmentations for errors and manually marked the location of Bruch's membrane opening (BMO). Custom software (Matlab, Mathworks) calculated the global TRT in an annulus extending from the BMO to a distance of 250 µm, as previously described (Laurie, Lee, et al., 2019; Macias et al., 2020; Patel et al., 2018). Circumpapillary retinal nerve fiber layer (RNFL) thickness and choroidal thickness were quantified from 6º‐radius circular scans centered on the ONH. Automated ILM, RNFL, and BM segmentations were corrected as needed, and the chorioscleral interface was manually delineated. RNFL thickness was quantified automatically using Heidelberg Eye Explorer software (Heidelberg Engineering) and choroidal thickness was quantified using custom Matlab programs. For all OCT measures, the final data represent the average values from two readers; these values were required to be within 10% of each other.

Two readers independently identified the formation of chorioretinal folds in subjects by reviewing all OCT images. These folds were classified as peripapillary wrinkles (closely spaced concentric or spiral folds confined to the RNFL and contained to within half a disc diameter of the ONH), retinal folds (periodic surface or intraretinal undulations located more than half a disc diameter from the ONH), and choroidal folds (undulations within the retinal pigment epithelium/BM complex), as described in the OCT sub‐study of the Idiopathic Intracranial Hypertension (IIH) Treatment Trial (Sibony et al., 2015).

### 2.5 Intraocular pressure

Intraocular pressure (IOP) was measured as the average of triplicate readings using the Icare PRO rebound tonometer (Icare) on BDC‐6, HDT2, HDT15, HDT31, HDT45, HDT58, R+6, and R+11. As with OCT data collection, IOP measures were obtained in seated and supine postures before HDTBR, in the 6º HDT posture during HDTBR, and in the supine posture after HDTBR.

### 2.6 Optical biometry and refractive error

Ocular globe flattening can occur during spaceflight, leading to hyperopic shifts in refractive error (Lee et al., 2020; Mader et al., 2011). Therefore, we investigated whether similar changes occur during 60 days of HDTBR. Optical biometry was performed using the IOLMaster 500 (Carl Zeiss Meditec AG) between BDC‐11 and BDC‐3 and between R+6 and R+9 in the seated posture to detect changes in axial length, anterior chamber depth, and corneal curvature. Cycloplegic refraction was performed on BDC‐12 and R+6 to determine whether a shift in refractive error had occurred.

### 2.7 Standard automated perimetry

On BDC‐12 and R+0, visual function was assessed at 53 predefined locations within the visual field (30º) using the Fast Threshold strategy of the OCULUS Twinfield perimeter (OCULUS, Inc.). The changes in mean deviation and pattern standard deviation were calculated.

### 2.8 Statistical analysis

Linear mixed effect methods were used to evaluate changes across outcome variables (compared to values collected in the baseline seated condition) and between experimental groups at each time point (each AG group compared to the control group). Time, group, and/or posture, and their interactions, were considered fixed effects in all analyses, whereas the eye (left vs. right) and/or repeated measurements by different analyzers were considered random effects. A separate regression was constructed for each outcome. Robust standard errors, calculated through a classic sandwich adjustment, were used to address the variances of different measurements across time points. In cases of significant *F*‐tests for time and group/position interactions, a Bonferroni adjustment of the *p*‐value was used account for multiple comparisons. All analyses were conducted in SAS, version 9.4 (SAS Institute) using the GLIMMIX procedure, including an LSMEANS statement for the pairwise comparisons. *P* < 0.05 was considered significant. Least squared means are presented. Unless otherwise indicated, the data described represent the average of both eyes. We previously modeled the precision to detect physiologically relevant changes in TRT that are beyond those changes that may occur based on normal day‐to‐day subject variability, acute posture changes, or differences based on different readers segmenting the images. Using data collected in seven subjects, studied ~4 months apart, in different postures, and analyzed by two readers, we determined that to quantify the earliest signs of optic disc edema from OCT images, the change in TRT would need to be greater than 19.4 µm (Laurie, Lee, et al., 2019).

## 3 RESULTS

### 3.1 Optic disc edema developed in all groups during HDTBR

**FIGURE 1:**
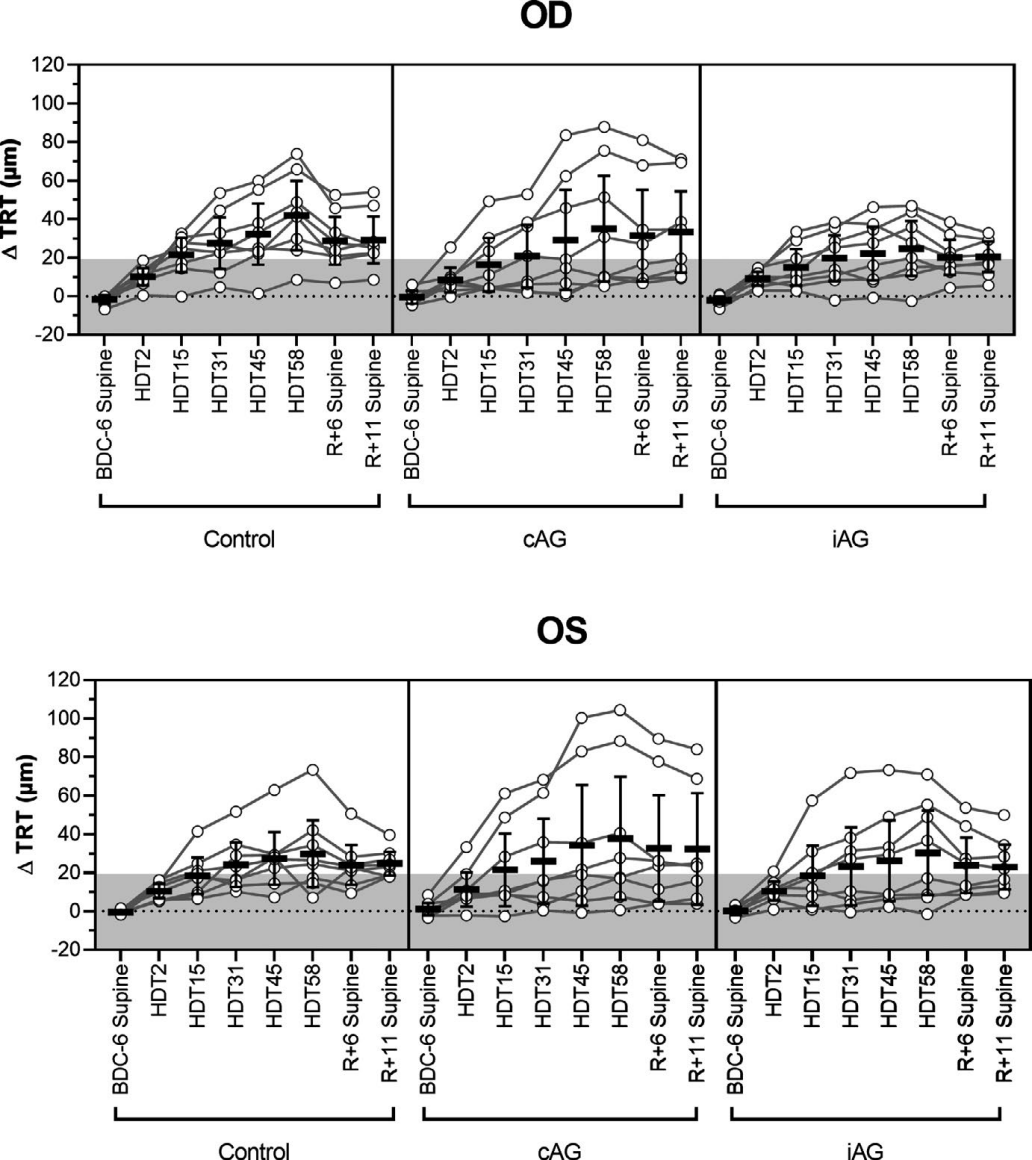
Optic disc edema is induced by strict 6º HDTBR but not mitigated by artificial gravity. The change in total retinal thickness (TRT) from BDC‐6 seated baseline at each time point for the right (OD) and left (OS) eyes of control (*n* = 8), continuous artificial gravity (cAG, *n* = 8), and intermittent artificial gravity (iAG, *n* = 8) subjects. Data for BDC‐6, R+6, and R+11 are presented for the supine posture. TRT increased with HDTBR duration for all three groups and did not return to baseline values by R+11. No differences between either experimental group and the control group were evident, indicating that the 30‐min artificial gravity protocols had no effect on TRT at any phase of the study. Circles represent individual subject data, lines connect data for individual subjects, horizontal bars represent the mean value across subjects, error bars represent the 95% CI, and the shaded area represents the predefined range (±19.4 µm) of normal day‐to‐day variation. Statistical significance for each time point is indicated in Table 2. HDT, head‐down tilt; BDC, baseline data collection; R, recovery

At seated baseline (BDC‐6), the mean TRT in the control group was 409.2 µm (95% CI, 392.4–426.0 µm), which was not different from the cAG group (400.3 µm, 95% CI, 381.7–418.9 µm, *p* = 0.97) or the iAG group (403.3 µm, 95% CI, 388.8–417.8 µm, *p* > 0.99). By HDT58, TRT increased in the control group by a mean of 35.9 µm (95% CI, 19.9–51.9 µm, *p *< 0.0001), in the cAG group by 36.5 µm (95% CI, 4.7–68.2 µm, *p* = 0.01), and in the iAG group by 27.6 µm (95% CI, 8.8–46.3 µm, *p* = 0.0005; Figure 1). We observed that 7 control subjects (12 eyes), 4 cAG subjects (8 eyes), and 5 iAG subjects (9 eyes) showed increases in TRT >19.4 µm, indicating subtle signs of optic disc edema. During HDTBR, increases in TRT in the cAG and iAG groups were not significantly different from the control group at any time point (cAG: *p* > 0.99; iAG: *p* > 0.61). After HDTBR on R+11, TRT remained increased relative to the pre‐HDTBR seated baseline by 27.0 µm (95% CI, 18.9–35.1 µm, *p* < 0.0001) for the control group, 32.5 µm (95% CI, 7.8–57.3 µm, *p* = 0.003) for the cAG group, and 21.8 µm (95% CI, 12.3–31.3 µm, *p* < 0.0001) for the iAG group. One subject (S1) from the iAG group had previously completed a separate 30‐day strict 6° HDTBR study (Laurie, Lee, et al., 2019); in the present study, the increase in TRT (26.1 µm) on HDT31 for this individual was ~2.5 times greater than on HDT30 in the prior study. No group showed significant changes in TRT with acute seated to supine posture changes at baseline (Control: *p* = 0.40; cAG: *p* > 0.99; iAG: *p* > 0.99, Table 2).

**TABLE 2:** Ocular measures before, during, and after HDTBR

		BDC−6 seated	BDC−6 supine	HDT2	HDT15	HDT31	HDT45	HDT58	R+6 supine	R+11 supine
TRT (µm)	Control	409.2 *392*.*4*–*426*.*0*	408.1 *391*.*0*–*425*.*2*	419.5*** *400*.*5*–*438*.*5*	429.2*** *408*.*8*–*449*.*6*	435.0*** *416*.*4*–*453*.*7*	439.1*** *420*.*0*–*458*.*2*	445.1*** *424*.*9*–*465*.*3*	435.6*** *417*.*8*–*453*.*3*	436.2*** *419*.*8*–*452*.*6*
	cAG	400.3 *381*.*7*–*418*.*9*	400.6 *381*.*6*–*419*.*5*	410.2** *388*.*3*–*432*.*0*	419.3* *392*.*4*–*446*.*1*	423.7* *395*.*8*–*451*.*7*	432.1* *398*.*2*–*465*.*9*	436.8* *401*.*5*–*472*.*1*	432.4* *400*.*2*–*464*.*6*	432.9** *402*.*2*–*463*.*5*
	iAG	403.3 *388*.*8*–*417*.*8*	402.3 *388*.*2*–*416*.*4*	413.1*** *398*.*6*–*427*.*5*	420.0** *404*.*3*–*435*.*8*	424.8** *408*.*7*–*441*.*0*	427.5** *409*.*7*–*445*.*3*	430.9*** *412*.*5*–*449*.*2*	425.3*** *409*.*1*–*441*.*5*	425.0*** *410*.*4*–*439*.*6*
RNFL Thickness (µm)	Control	103.1 *95*.*4*–*110*.*8*	103.0 *95*.*2*–*110*.*8*	103.2 *94*.*8*–*111*.*5*	104.0 *96*.*0*–*112*.*0*	103.7 *95*.*6*–*111*.*8*	104.6* *96*.*7*–*112*.*4*	105.8*** *97*.*9*–*113*.*6*	105.7*** *97*.*9*–*113*.*5*	106.3*** *98*.*3*–*114*.*2*
	cAG	100.4 *93*.*4*–*107*.*5*	99.9 *92*.*8*–*106*.*9*	100.0 *92*.*9*–*107*.*1*	100.3 *93*.*0*–*107*.*5*	101.1 *93*.*5*–*108*.*6*	101.7 *93*.*7*–*109*.*7*	102.3 *94*.*2*–*110*.*4*	103.4* *95*.*7*–*111*.*2*	103.5* *95*.*8*–*111*.*2*
	iAG	101.2 *95*.*6*–*106*.*8*	101.0 *95*.*4*–*106*.*5*	100.8 *95*.*4*–*106*.*2*	101.2 *96*.*0*–*106*.*3*	101.4 *96*.*5*–*106*.*3*	101.8 *96*.*6*–*107*.*0*	102.2 *97*.*3*–*107*.*1*	103.0* *98*.*0*–*108*.*0*	102.7** *97*.*8*–*107*.*7*
Choroidal Thickness (µm)	Control	235.9 *204*.*9*–*267*.*0*	239.9 *208*.*2*–*271*.*6*	240.2 *209*.*3*–*271*.*1*	239.3 *208*.*3*–*270*.*3*	242.3 *209*.*7*–*274*.*9*	241.6 *207*.*8*–*275*.*3*	242.5 *210*.*0*–*275*.*0*	225.9 *194*.*5*–*257*.*2*	222.3* *188*.*5*–*256*.*0*
	cAG	209.3 *181*.*6*––*237*.*0*	212.4 *182*.*8*–*241*.*9*	210.9 *180*.*9*–*240*.*9*	214.3 *183*.*6*–*245*.*0*	212.5 *181*.*6*–*243*.*3*	209.2 *179*.*2*–*239*.*3*	209.6 *179*.*7*–*239*.*4*	205.4 *176*.*4*–*234*.*5*	204.8 *176*.*0*–*233*.*6*
	iAG	239.6 *208*.*1*–*271*.*1*	244.1*** *212*.*1*–*276*.*1*	241.7 *207*.*1*–*276*.*3*	244.4 *210*.*0*–*278*.*8*	241.9 *202*.*1*–*281*.*7*	239.2 *201*.*2*–*277*.*2*	240.0 *202*.*1*–*277*.*8*	227.9 *190*.*7*–*265*.*0*	223.6** *187*.*4*–*259*.*8*
IOP (mmHg)	Control	13.6 *12*.*2*–*15*.*1*	15.5 *13*.*9*–*17*.*1*	16.2** *15*.*2*–*17*.*3*	15.3 *14*.*5*–*16*.*1*	14.9 *14*.*1*–*15*.*7*	15.2* *13*.*8*–*16*.*7*	17.0*** *15*.*2*–*18*.*9*	15.4 *14*.*0*–*16*.*7*	15.3 *14*.*6*–*16*.*0*
	cAG	14.7 *13*.*2*–*16*.*2*	15.8 *14*.*1*–*17*.*5*	16.8*** *15*.*6*–*17*.*9*	16.3 *15*.*2*–*17*.*4*	16.5†† *16*.*0*–*17*.*0*	15.9 *14*.*5*–*17*.*2*	16.9** *16*.*0*–*17*.*7*	16.2 *15*.*1*–*17*.*4*	16.8*** *15*.*5*–*18*.*1*
	iAG	14.4 *13*.*9*–*14*.*8*	16.4*** *15*.*5*–*17*.*2*	16.2 *14*.*5*–*17*.*9*	15.4* *14*.*4*–*16*.*5*	16.0** *15*.*0*–*17*.*1*	16.7*** *15*.*7*–*17*.*7*	15.7 *14*.*2*–*17*.*1*	16.9*** *15*.*6*–*18*.*1*	15.7*** *15*.*1*–*16*.*2*

Values represent the mean and 95% CI (italics). Significant difference in comparison to BDC‐6 seated: *p < *0*.*05*, **p < *0*.*01*, ***p < *0*.*001*. Significant difference in comparison to the control group: ††*p* < *0*.*01*.

Abbreviations: AG, continuous artificial gravity; BDC, baseline data collection; HDT, head‐down tilt; iAG, intermittent artificial gravity; IOP, intraocular pressure; R+, recovery day; RNFL, retinal nerve fiber layer; TRT, total retinal thickness.

Compared to values for seated baseline (BDC‐6), the mean RNFL thickness measured from the 6°‐radius circle scan was not significantly different at any time point during HDTBR, with the exceptions of a 1.5 µm (95% CI, 0.0–2.9 µm, *p* = 0.0497) increase at HDT45 and a 2.7 µm (95% CI, 0.9–4.4 µm, *p* = 0.0003) increase at HDT58 for the control cohort (Table 2). Acute changes in posture at baseline did not affect RNFL thickness (Control: *p* > 0.99; cAG: *p* = 0.71; iAG: *p* > 0.99).

### 3.2 Chorioretinal folds developed in all groups during HDTBR

**FIGURE 2:**
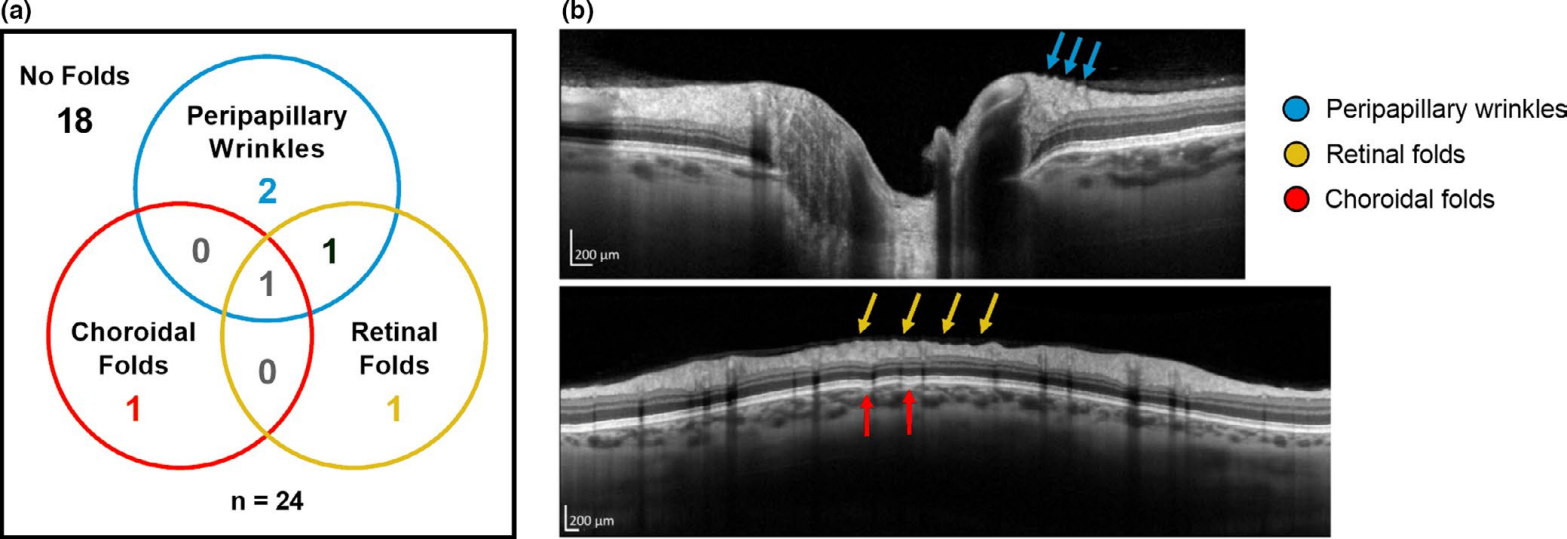
Chorioretinal folds can develop during HDTBR. (a) Six of 24 subjects developed chorioretinal folds (peripapillary wrinkles, retinal folds, and/or choroidal folds) during HDTBR. Four subjects presented with a single subtype, 1 subject presented with both retinal and choroidal folds in the same eye, and 1 subject presented all three subtypes in the same eye. (b) OCT images show examples of peripapillary wrinkles (top, cyan arrows), retinal folds (bottom, yellow arrows), and choroidal folds (bottom, red arrows) in 1 HDTBR subject (Subject C). Scale bars represent 200 µm

Choroidal folds, retinal folds, and/or peripapillary wrinkles were observed in 6 of the 24 of study participants on HDT58, with incidence in each experimental group (Figure 2). Four subjects developed a single type of fold, one subject developed retinal and choroidal folds in the same eye, and one subject developed all three types in the same eye. Only the subject who presented with isolated retinal folds was affected bilaterally. All chorioretinal folds that developed were still present on R+11, except for one eye in which isolated peripapillary wrinkles had resolved (Subject N).

### 3.3 Choroidal thickness did not increase during HDTBR

**FIGURE 3:**
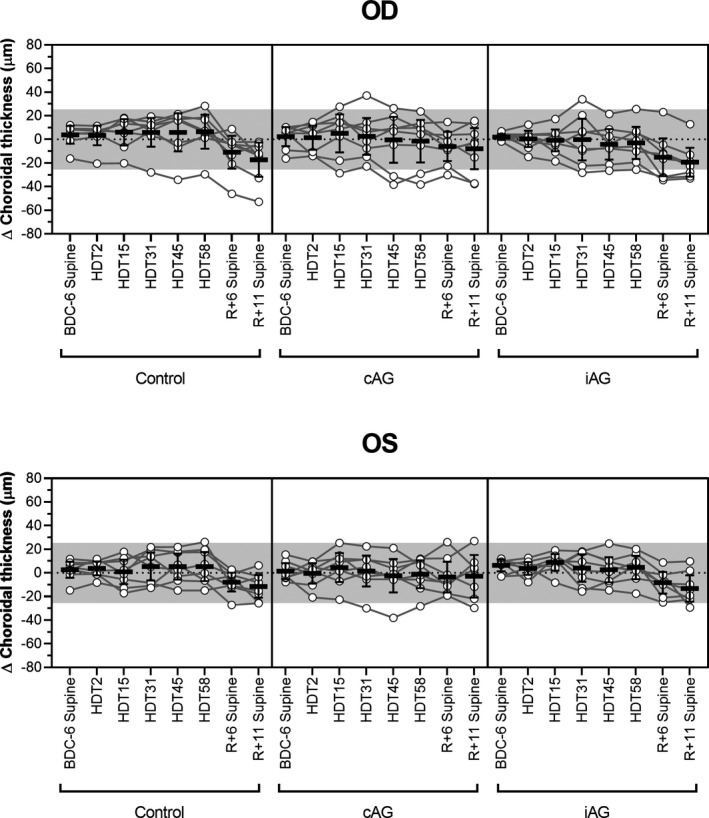
Choroidal thickening is not induced by HDTBR. No significant change in mean choroidal thickness, as compared to seated baseline values (BDC‐6), was detected at any time point during HDTBR. During recovery after HDTBR (R+6 and R+11), the choroid thinned relative to baseline. No differences were detected between either experimental group and the control group for any phase of the study. Circles represent individual subject data, lines connect data for individual subjects, horizontal bars represent the mean value across subjects, error bars represent the 95% CI, and the shaded area represents the predefined range (±25.5 µm) of normal day‐to‐day variation. Statistical significance for each time point is indicated in Table 2. HDT, head‐down tilt; BDC, baseline data collection; R, recovery. OD, right eye; OS, left eye

For all groups, choroidal thickness during the HDTBR phase of the study was equivalent to the baseline values collected in the seated posture (Control: *p* > 0.46; cAG: *p* > 0.99; iAG: *p* > 0.40), and neither AG protocol affected this variable (cAG: *p *> 0.29; iAG: *p* > 0.99, Figure 3). Relative to the baseline values, choroidal thickness during the recovery phase on R+11 decreased by 13.6 µm (95% CI, 1.8–25.5 µm, *p* = 0.013) for the control group and by 16.0 µm (95% CI, 4.2–27.8 µm, *p* = 0.0018) for the iAG group. An acute change from the seated to supine position on BDC‐6 resulted in a statistically significant increase in choroidal thickness for the iAG group (4.5 µm, 95% CI, 1.4–7.6 µm, *p* = 0.0006), but no significant change was detected for the control (*p* = 0.53) or cAG groups (*p* > 0.99).

### 3.4 Intraocular pressure was mildly elevated in supine and HDT postures

IOP remained within the normal clinical range (Wang et al., 2018) throughout the duration of the study, with the variation generally being less than the magnitude of typical diurnal changes (Liu et al., 2003). In general, transition from a seated to a supine posture before HDTBR resulted in a 1–2 mmHg increase in IOP; however, this change was only significant for the iAG group (2.0 mmHg, 95% CI, 0.9–3.1 mmHg, *p *< 0.0001). At various time points during HDTBR, all three groups exhibited significant increases in IOP from the BDC‐6 seated baseline values (Table 2). IOP for the cAG group only differed from the control group on HDT31, at which point it was 1.6 mmHg greater (95% CI, 0.5–2.7 mmHg, *p* = 0.002), and IOP for the iAG group did not significantly differ from controls at any time point (*p* > 0.18).

### 3.5 Neither ocular biometry measures nor visual function were altered by HDTBR

No changes in axial length, anterior chamber depth, or corneal curvature were detected in any group, with the exception of a small decrease (0.05 mm, 95% CI, 0.003–0.093 mm, *p* = 0.04) in anterior chamber depth for the control group after HDTBR (Table 3). Similarly, no change in cycloplegic refractive error was observed (Control: *p* = 0.19; cAG: *p* = 0.56; iAG: *p* = 0.26), and no change in mean deviation (Control: *p* = 0.38; cAG: *p* = 0.95; iAG: *p* = 0.45) or pattern standard deviation (Control: *p* = 0.61; cAG: *p* = 0.51; iAG: *p* = 0.13) was detected by automated perimetry after HDTBR.

**TABLE 3:** Refractive error, ocular biometry, and visual field before and after HDTBR

Measurement	Condition	Pre‐HDTBR	Post‐HDTBR	*p*
SE Refractive Error (D)	Control	−0.18 −*1*.*27* to +*0*.*91*	+0.09 −*1*.*00* to +*1*.*19*	0.19
	cAG	−0.26 −*0*.*98* to +*0*.*46*	−0.31 −*0*.*91* to +*0*.*30*	0.56
	iAG	−0.38 −*1*.*23* to +*0*.*48*	−0.47 −*1*.*44* to +*0*.*50*	0.26
Axial Length (mm)	Control	23.70 *23*.*05*–*24*.*36*	23.71 *23*.*03*–*24*.*38*	0.74
	cAG	24.06 *23*.*54*–*24*.*57*	24.06 *23*.*54*–*24*.*59*	0.77
	iAG	23.75 *23*.*33*–*24*.*17*	23.78 *23*.*34*–*24*.*21*	0.12
Anterior Chamber Depth (mm)	Control	3.64 *3*.*44*–*3*.*83*	3.59 *3*.*38*–*3*.*79*	0.04
	cAG	3.62 *3*.*40*–*3*.*85*	3.63 *3*.*40*–*3*.*86*	0.63
	iAG	3.57 *3*.*43*–*3*.*71*	3.55 *3*.*39*–*3*.*71*	0.39
Avg. Corneal Curvature (mm)	Control	7.74 *7*.*62*–*7*.*87*	7.72 *7*.*60*–*7*.*85*	0.10
	cAG	7.87 *7*.*70*–*8*.*04*	7.88 *7*.*71*–*8*.*05*	0.28
	iAG	7.82 *7*.*67*–*7*.*97*	7.82 *7*.*68*–*7*.*97*	0.94
Visual Field MD (dB)	Control	+0.62 −*0*.*46* to +*1*.*69*	+1.00 −*0*.*15* to +*2*.*15*	0.38
	cAG	+0.24 −*0*.*46* to +*0*.*93*	+0.26 −*0*.*48* to +*1*.*00*	0.95
	iAG	−0.39 *−1.41 to +0.63*	−0.12 −*0*.*95* to +*0*.*71*	0.45
Visual Field PSD (dB)	Control	7.81 *4*.*38*–*11*.*24*	7.02 *5*.*33*–*8*.*71*	0.61
	cAG	7.98 *2*.*48*–*13*.*48*	6.76 *4*.*39*–*9*.*13*	0.51
	iAG	6.28 *2*.*17*–*10*.*40*	9.07 *3*.*83*–*14*.*31*	0.13

Ophthalmological measurements collected before and after head‐down tilt bed rest (HDTBR). Values represent the mean and 95% CI (italics). No significant differences were detected between control and either AG group for any parameter.

Abbreviations: cAG, continuous artificial gravity; iAG, intermittent artificial gravity; MD, mean deviation; PSD, pattern standard deviation; SE, spherical equivalent.

### 3.6 Potential relationships between chorioretinal folds and changes in other ocular parameters

**FIGURE 4:**
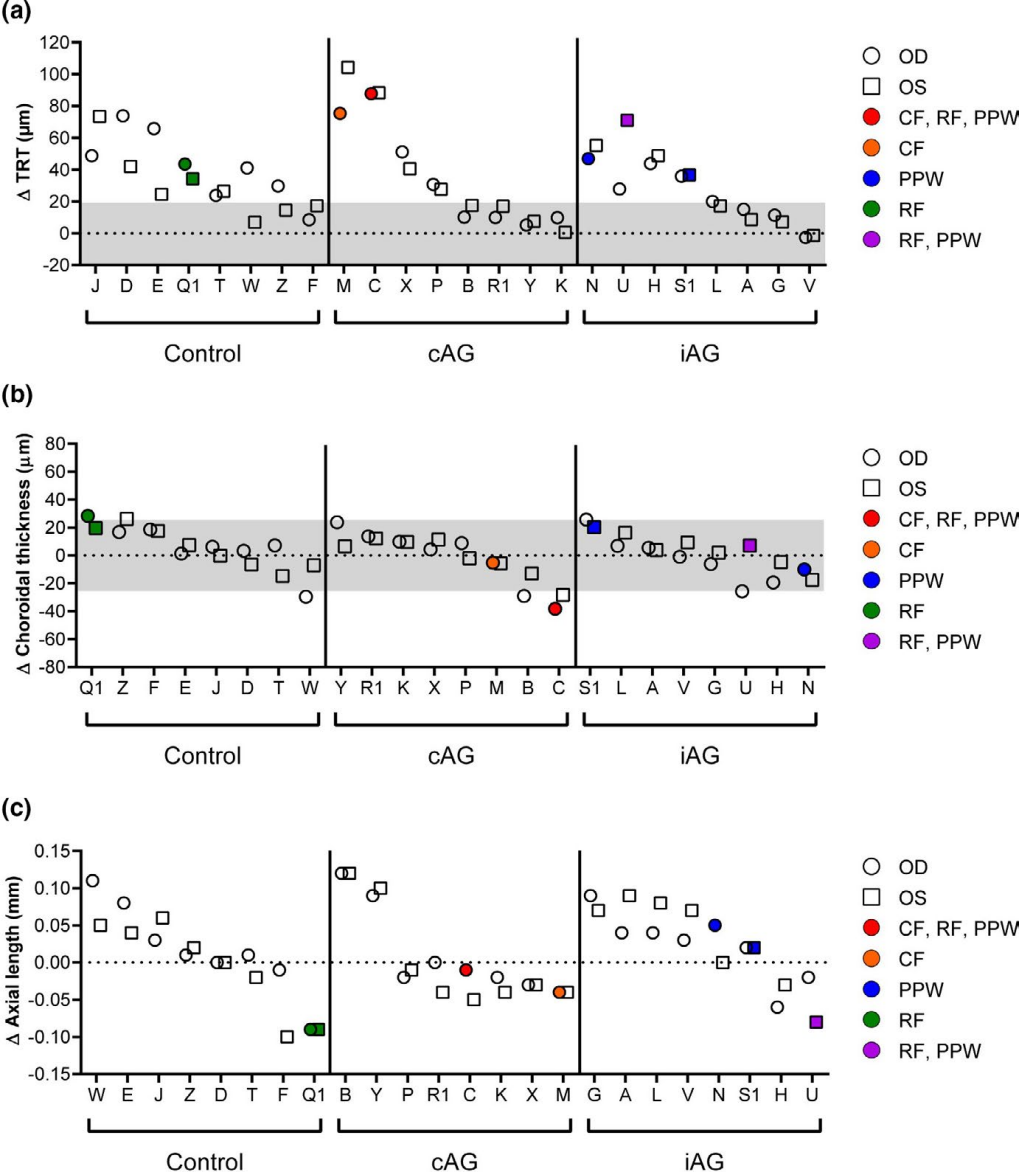
Relationship between chorioretinal folds and changes in other ocular parameters on HDT58. (a) Chorioretinal folds only developed in eyes that showed subtle signs of optic disc edema based on increases in total retinal thickness (TRT) exceeding the predefined 19.4 µm threshold (shaded gray area), and the two instances of choroidal folds occurred in the 2 subjects who had the greatest increases in TRT. Only the subject with isolated retinal folds was affected bilaterally. (b) No consistent pattern was observed between chorioretinal fold development and changes in choroidal thickness. (c) The two subjects with retinal folds had decreases in axial length. OD, right eye (circles); OS, left eye (squares); CF, choroidal folds; RF, retinal folds; PPW, peripapillary wrinkles

To determine whether the type of chorioretinal fold that developed was related to other structural changes around the ONH, the fold status of individual eyes was compared to changes in TRT and choroidal thickness at HDT58. For all eyes that formed folds, the increase in TRT was >24 µm (Figure 4A). The two subjects with choroidal folds exhibited the greatest increases in TRT (Subjects M and C). No relationship was apparent between chorioretinal fold development and changes in choroidal thickness.

Substantial decreases in IOP and/or axial length have been associated with the development of chorioretinal folds in patient populations (Costa & Arcieri, 2007; Williams et al., 2015; Yalçındağ et al., 2011). Therefore, we assessed whether similar changes in these parameters were associated with the development of chorioretinal folds during HDTBR. For the seven eyes that developed chorioretinal folds, the average change in IOP during HDTBR was an increase of 1.7 mmHg (range, 0.8–2.8 mmHg) relative to the seated baseline value; IOP for each of these eyes remained within a normal range. The mean decrease in axial length for the seven eyes that developed chorioretinal folds was small (−0.03 mm, range, −0.09–0.05 mm); plots of axial length change versus type of fold development for individual subjects are shown in Figure 4C. The two subjects who had retinal folds and the greatest decreases in axial length (‘Q1’: right eye (OD) and left eye (OS); ‘U’: OS) had hyperopic shifts in refractive error (+0.25 D and +0.63 D, +0.25 D, respectively) that were generally consistent with the axial length measurement.

## 4 DISCUSSION

This study investigated whether a 60‐day exposure to strict 6° HDTBR, a spaceflight analog, would produce ocular structural changes consistent with SANS in healthy subjects and whether a daily application of AG by short‐arm centrifugation could prevent or attenuate these changes. Similar to a previous study in which healthy subjects were exposed to strict HDTBR in a mildly hypercapnic environment and to multiple studies that have demonstrated ocular findings in astronauts during and after long‐duration spaceflight, the increases in TRT reported in this study suggest a subtle development of optic disc edema. Although chorioretinal folds have been documented in astronauts during long‐duration spaceflight (Mader et al., 2011), the present study is the first to show that chorioretinal folds can develop in HDTBR subjects. Furthermore, a daily 30‐min exposure to either continuous or intermittent AG by centrifugation did not lessen ocular structural changes that were generated by HDTBR.

This study confirms our previous report that strict HDTBR can induce the development of optic disc edema. Although the mean increase in TRT on HDT31 of the present study was approximately half of that observed in our previous 30‐day strict HDTBR study (Laurie, Lee, et al., 2019), a similar proportion of subjects in both studies had increases in TRT that indicate subtle signs of optic disc edema; this effect was not observed in subjects during a 70‐day HDTBR study that did not use the strict form of head‐down tilt positioning (Taibbi et al., 2016). The mean increase in TRT spanned a similar range to that reported in astronauts during spaceflight at similar time points (Macias et al., 2020). These data confirm that strict HDTBR induces optic disc edema without a mild elevation in ambient CO_2_ levels and demonstrate for the first time that HDTBR leads to the development of chorioretinal folds, which were not observed in the previous 30‐day study (Laurie, Lee, et al., 2019). The edema that develops within 60 days of strict HDTBR is mild, as circumpapillary RNFL thickness remained within normal levels.

One subject in the present study participated in our previous 30‐day strict 6° HDTBR experiment (Laurie, Lee, et al., 2019). For this individual, the increase in TRT on HDT31 of the present study was more than twice the increase observed on HDT30 in the previous study, and TRT continued to increase in this individual through HDT58. It is unknown if the prior exposure to the sustained fluid shift caused the optic nerve head and adjacent retina to be more susceptible to developing optic disc edema when exposed to the chronic headward fluid shift in the current study or if daily exposure to AG (iAG) contributed to the greater increase in TRT. Further research is needed to understand whether repeated exposure to a chronic headward fluid shift increases the risk of developing optic disc edema.

Daily 30‐min exposures to continuous or intermittent AG via centrifugation did not prevent the development of optic disc edema. Whether a longer duration AG exposure and/or greater g‐forces at the level of the eye (>0.3 g) are needed to reverse the impact of the chronic headward fluid shift requires further investigation. We have hypothesized that elevated venous pressure (Lawley et al., 2017; Marshall‐Goebel, Laurie, et al., 2019) may increase capillary filtration in the prelaminar region of the optic nerve head, which lacks blood‐brain barrier markers (Hofman et al., 2001), and thereby contribute to the development of optic disc edema (Laurie, Lee, et al., 2019; Stenger et al., 2019; Greenwald et al., 2021, *Online ahead of print*). If this is true, the 30‐min AG exposure may not have been long enough to allow for a reabsorption of extravascular fluid within the retinal microcirculation at the optic nerve head.

Structural modeling data suggest that spaceflight‐induced choroidal thickening (Macias et al., 2020; Mader et al., 2011; Greenwald et al., 2021, *Online ahead of print*) may increase strain at the optic nerve head, thereby contributing to optic disc edema (Feola et al., 2018). Although studies involving acute headward fluid shifts, including a recent study using three days of strict 6º HDTBR, have reported statistically significant changes in choroidal thickness or area (Balasubramanian et al., 2018; Lawley et al., 2020; Shinojima et al., 2012), the magnitude of the change observed in the present study and in our previous strict HDTBR study (Laurie, Lee, et al., 2019) were small relative to the reported changes in astronauts during spaceflight (Macias et al., 2020; Mader et al., 2017). Therefore, the relative lack of change in the choroid observed during the development of subtle signs of optic disc edema, as indicated by increases in TRT, suggests that choroidal thickening is not required for the development of edema in this spaceflight analog. Whether this is also true in astronauts who develop SANS and have increased choroidal thickness is unknown. As we also observed in our previous strict HDTBR study (Laurie, Lee, et al., 2019), during the post‐bed rest period the choroid can become thinner than it was at baseline; the reason and implications for this effect require further investigation. Previous work suggests that a change in choroidal thickness greater than 25.5 µm is needed to exceed the normal day‐to‐day variation that can occur while a single posture is maintained (Marshall‐Goebel et al., 2021, *Online ahead of print*).

The development of chorioretinal folds observed during the present strict HDTBR experiment further supports that this analog of spaceflight‐induced weightlessness reliably models aspects of SANS, as chorioretinal folds have been documented in a subset of astronauts during long‐duration spaceflight (Mader et al., 2011, 2013). Given that at least one subject in each of the three groups developed chorioretinal folds, a daily 30‐min exposure to AG using the present centrifugation conditions is not sufficient to prevent the development of this structural change. We investigated whether the chorioretinal folds observed (seven eyes total) were related to other HDTBR‐induced ocular changes, although the trends should be interpreted with caution given the small sample size. A relationship between chorioretinal folds and optic disc edema appears possible because all eyes with chorioretinal folds also had increases in TRT that were above the normal variability threshold; however, the reason why many retinas with substantial changes in TRT did not develop folds, including the fellow retinas of those that did, is unclear. Conversely, during our previous strict HDTBR study, we did not detect chorioretinal folds in subjects who had greater increases in TRT than those presented here (Laurie, Lee, et al., 2019). Spaceflight‐induced choroidal thickening has been hypothesized to facilitate choroidal fold development; however, evidence for this effect was not observed in the present study. The cause of the individual variability in chorioretinal fold development may depend on the tissue properties of the individual eye (Mader et al., 2011). It has been hypothesized that spaceflight‐induced globe flattening (Macias et al., 2020) contributes to chorioretinal folds because of a decrease in surface area of the posterior globe (Jacobson, 1995; Marshall‐Goebel, Damani, et al., 2019; Marshall‐Goebel, Laurie, et al., 2019); we did not observe changes in axial length indicative of globe flattening in our subjects, despite the presence of chorioretinal folds.

The formation of chorioretinal folds caused by terrestrial disease has been linked to changes in IOP and intracranial pressure (ICP). Studies of hypotony and IOP‐lowering drainage procedures have associated fold development with decreased IOP and low scleral rigidity (Fannin et al., 2003; Osigian et al., 2018; Thomas et al., 2015). However, given that IOP did not decrease in the present study, low IOP cannot explain chorioretinal fold development in HDTBR subjects. Similarly, astronauts do not develop ocular hypotony during spaceflight (Draeger et al., 1995; Stenger et al., 2017) (Greenwald et al., 2021, *Online ahead of print*). In a population of patients with optic disc edema resulting from IIH, only ~10% had choroidal folds, whereas ~45% had retinal folds or peripapillary wrinkles (Sibony et al., 2015). This suggests that elevated ICP, along with substantial thickening of the peripapillary retina, is not sufficient to cause chorioretinal folds in all subjects. Although direct measurements of ICP have neither been collected during spaceflight nor during HDTBR, direct measurements collected during brief periods of parabolic flight or during 24 h of HDT do not indicate pathologically elevated ICP (Lawley et al., 2017). A mild elevation in ICP likely occurred in our subjects due to the lack of a daily upright posture‐induced lowering of ICP. Whether a mild elevation in ICP would be sufficient to cause the retinal structural changes observed here, and whether the types of retinal structural changes resemble those reported in the IIH patients, remain open questions.

This study had several limitations. We did not observe the small decrease in axial length that develops in astronauts, highlighting a possible difference between the ocular changes caused by HDTBR and those resulting from spaceflight. Due to testing requirements for other AGBRESA‐related experiment protocols not reported here (e.g., MRI), subjects spent a total of 9 h in the 0° supine posture over the 60 days of HDTBR, in addition to the time in the supine posture during AG. Additionally, using a short‐arm centrifuge to apply 1 g at the center of mass resulted in only ~0.3 g at the level of the eye. If AG is to be considered as a SANS countermeasure in the future, it may be beneficial to extend the radius of the centrifuge arm to increase the g‐force at the level of the eye (e.g., long‐arm centrifuge). These HDTBR studies are labor intensive and require specialized human research facilities, therefore future study designs with shorter duration may be warranted to efficiently test countermeasures.

In summary, we show that strict 6º HDTBR can produce both chorioretinal folds and subtle signs of optic disc edema in healthy test subjects. Moreover, these findings occurred without subjects being exposed to elevated CO_2_ during HDTBR. These observations further support the use of this analog of spaceflight‐induced weightlessness for characterizing the development of findings similar to those that occur in SANS and for testing potential SANS countermeasures. The magnitude of the optic disc edema was similar to that reported in astronauts, but mild in comparison to optic disc edema associated with terrestrial diseases. No changes in refractive error or perimetry developed. Our first attempt to test AG as a potential SANS countermeasure, by using a daily 30‐min exposure to centrifugation, was unable to mitigate the development of the ocular changes. Future countermeasure investigations should target a longer duration and/or greater magnitude of exposure that induces a greater fluid shift reversal at the level of the eye to prevent the development of optic disc edema and chorioretinal folds.

## CONFLICT OF INTEREST

The authors declare that they have no competing interests.

## AUTHOR CONTRIBUTIONS

Data collection occurred at the German Aerospace Center in Cologne, Germany. SSL, KMG, SMCL, CS, BRM, and EMB contributed to the conception and/or design of the study. CS contributed to acquisition of the data. SSL, SHG, KMG, LPP, CS, HSH, BRM, and EMB contributed to the analysis and/or interpretation of the data. SSL, SHG, LPP, and BRM drafted the manuscript. All authors provided inputs to the manuscript and approved the final version. All authors have agreed to be accountable for all aspects of the work and ensure that questions related to the accuracy or integrity of any part of the work have been appropriately investigated and resolved. All persons who have been designated as authors qualify for authorship and all those who qualify for authorship are listed.

## Supporting information



Supplementary Material

## Data Availability

The data that support the findings of this study are available upon request from NASA’s Life Sciences Data Archive (LSDA, https://lsda.jsc.nasa.gov/Request/dataRequest). The data are not publicly available due to privacy or ethical restrictions.
