# Supplementary material for: Optic disc edema and chorioretinal folds develop during strict 6° head‐down tilt bed rest with or without artificial gravity
Source: Physiol Rep. 2021 Aug 6;9(15):e14977. doi: 10.14814/phy2.14977 (PMC8343460; doi:10.14814/phy2.14977)
Supplement: Supplementary file 1 — Supplementary Material [file PHY2-9-e14977-s001.pdf]

National Aeronautics and Space Administration
Johnson Space Center
Human Exploration and Operations Mission Directorate
Human Research Program
Houston, TX 77058

Human Exploration Research Opportunities (HERO)

NNJ15ZSA001N-AGBR

Appendix G

Physiological and Behavioral Responses in Humans to Intermittent Artificial Gravity during Bed Rest

Step-1 Response Period: December 1, 2015 – January 4, 2016
Step-1 Proposals Due: January 4, 2016, 5 PM Eastern Time
Step-2 Response Period: January 5, 2016 – March 7, 2016
Step-2 Proposals Due: March 7, 2016, 5 PM Eastern Time
Estimated Step-2 Selection Announcement: June 2016

You must read and understand this solicitation in its entirety to prepare a competitive proposal. Key requirements are identified here:

- **The information and specific submission instructions in this NRA supersede that found in the NASA Guidebook for Proposers. Proposals that do not conform to the requirements in this NRA may be declared noncompliant and declined without review.**
- For Step-1 and Step-2 proposals: You and your organization must be registered with NSPIRES. Your proposal must be submitted by an authorized representative of your organization. All team members listed on the proposal must be registered with NSPIRES.
- For Step-1 and Step-2 proposals: Your specific aims must address the research emphases in this solicitation, and must be clearly outlined in the project description of your proposal.
- For Step-2 proposals: Proposers must identify the Human Research Roadmap (HRR) risks and gaps that are being addressed by their proposal (http://humanresearchroadmap.nasa.gov/)
- For Step-2 proposals: The length of the project description of the proposal cannot exceed 20 pages using standard (12 point) type.
- For Step-2 proposals: Investigators submitting a proposal in response to this solicitation, and whose most recent submission that included similar specific aims to any NASA or NSBRI sponsored research announcement was not accepted, must address prior review comments (2 pages maximum).
- Investigators resubmitting a proposal in response to this solicitation may only submit a proposal with similar hypothesis(es) and aims a total of three times (original submission plus two resubmissions). Significant changes must be made to the proposal hypothesis(es) and specific aims for consideration after the third attempt or the proposal will be declined without further review.
- For Step-2 proposals: If you have received past NASA or NSBRI supported research within the last four years, you must provide specifics (2 pages maximum) to the productivity of your research in a section separate from the project description.
- For Step-2 proposals: Your proposal must meet requirements of the Compliance Review section of this solicitation.
- For Step-2 proposals: Inclusion of the Analog Study Resource Worksheet.
- For Step-2 proposals: A thorough statistical section must be included which includes a power analysis for the estimate of sample size and the comparison of males and females unless compelling evidence is provided that shows that no sex differences are expected.
- Step-1 and Step-2 selection decision information can be accessed after the selection announcement date listed in this solicitation. After logging in, the PI selects the "Proposals" link, the "Submitted Proposals/NOIs" link, and then clicks on the proposal submitted to the solicitation identified above. The document(s) provided by NASA will be displayed under the heading "PI Information Package" located at the bottom of the "View Proposal" page.

Appendix G

A. Funding Opportunity Description

1. Introduction

This solicitation supports research that NASA is planning to conduct at the *:envihab* facility located in Cologne, Germany at the German Aerospace Center Institute for Aerospace Medicine (http://www.dlr.de/envihab/en/desktopdefault.aspx). The *:envihab* is a state of the art facility for conducting ground-based research in support of spaceflight. Proposals selected in response to this topic will be conducted in the *:envihab* bed rest module and will make use of the *:envihab* short-arm centrifuge. Subjects will undergo 60 days of 6° head-down tilt bed rest (HDBR). They will be ambulatory for two weeks prior to and after bed rest. This time will allow for baseline data collections and recovery after bed rest. During the bed rest phase subjects will be randomly assigned to one of three experimental groups (n=8 in each group; total n=24).

Experimental groups:

- **Group 1:** 6° HDBR with no centrifugation (control).
- **Group 2:** 6° HDBR with supine centrifugation at +1Gz at the heart for 30 minutes per day.
- **Group 3:** 6° HDBR with supine centrifugation at +1Gz at the heart for 6 bouts of 5 minutes per day (sessions 2-6 are separated by 5 minutes of rest).

Depending on the height of the subjects and their position relative to the rotational axis of the centrifuge, the rotation rate will range from 25 to 30 rpm. This rate corresponds to about +2Gz at the feet. Angular acceleration and deceleration during spin-up and spin-down of the centrifuge will not exceed $5°s^{-2}$. For more information on the :envihab short-arm centrifuge capabilities see the *:envihab Short Arm Centrifuge* document posted alongside of this solicitation on the NASA Solicitation and Proposal Integrated Review and Evaluation System (NSPIRES) solicitation download site (http://nspires.nasaprs.com/external/).

NASA will utilize a standardized set of measures to examine the effects of these bed rest conditions on multiple body systems. Investigators proposing to this solicitation will be able to utilize the standard measures in addition to proposing measures specific to their study. The intent is to include as many of the standard measures as possible. However, should conflicts arise between the standard measure and investigator's measure, the investigator's measure will have priority provided a sufficient rationale is presented in the proposal as to why the standard measure does not meet the study design. For further information on the bed rest platform and standard measures see the *Human Research Program Flight Analogs Project Information Package for Bed Rest Studies* posted alongside of this announcement at http://nspires.nasaprs.com/external/.

To be responsive to this research solicitation, proposed studies should lead to specific products that address at least one of the three specific objectives outlined in the HERO Overview document, section B.2, Goals and Objectives. The proposed studies should lead to new

knowledge within accepted scientific standards. Proposals should take into account the impact of sex, age, nutrition, stress, genetic predisposition, or sensitivity to other factors of importance. A thorough statistical section must be included, and must contain a power analysis for the estimate of sample size and the comparison of males and females unless compelling evidence is provided that shows that no sex differences are expected. Please see the Sample Size Specification Guidelines posted at http://nspires.nasaprs.com/external/ alongside this document for additional information about sample size calculations.

Proposers from the U.S. are strongly encouraged to include international partners from countries supporting space agencies that participate in the ISS Program. Proposals led by international partners can also be submitted. If you are an international researcher intending to submit a proposal, please read this document and then see the special additional instructions posted on NSPIRES alongside this appendix. Please note however, that grant support from NASA can only be provided to scientists from the U.S. Any non-U.S. participation must be supported by the country or agency sponsoring the non-U.S. proposer. Proposals involving non-U.S. participants must include a **letter from their country or space agency** stating that the proposal will be considered for funding once it passes the peer review process.

Proposals must be responsive to the research emphasis outlined below in order to be reviewed as significant to the goals of this solicitation. The proposed research approach must adhere to all constraints and guidelines outlined in this solicitation.

2. Research Emphasis

a. Physiological and Behavioral Responses in Humans to Intermittent Artificial Gravity during 60-Days of Head-down Tilted Bed Rest

PRD Risks:

- Risk of impaired control of spacecraft/associated systems and decreased mobility due to vestibular/sensorimotor alterations associated with spaceflight.
- Risk of orthostatic intolerance during re-exposure to gravity.
- Risk of cardiac rhythm problems.
- Risk of spaceflight-induced intracranial hypertension/vision alterations.
- Risk of impaired performance due to reduced muscle mass, strength and endurance.
- Risk of early onset osteoporosis due to spaceflight.
- Risk of adverse cognitive or behavioral conditions and psychiatric disorders.
- Risk of Performance Decrements and Adverse Health Outcomes Resulting from Sleep Loss, Circadian Desynchronization, and Work Overload.

IRP Gaps:

- **SM1**: Determine the changes in sensorimotor function over the course of a mission and during recovery after landing.
- **SM24**: Determine if the individual capacity to produce adaptive change (rate and extent)

in sensorimotor function to transitions in gravitational environments can be predicted with preflight tests and sensorimotor adaptability.

- **SM28:** Develop a sensorimotor countermeasure system integrated with current exercise modalities to mitigate performance decrements during and after spaceflight.
- **CV3:** Is orthostatic intolerance a potential hazard?
- **CV8:** Can manifestations of sub-clinical or environmentally induced cardiovascular diseases during spaceflight be predicted?
- **VIIP1:** We do not know the etiological mechanisms and contributing risk factors for ocular structural and functional changes seen in-flight and post-flight.
- **M6:** Develop pre-flight and in-flight evaluations to determine if muscle fitness standards are met.
- **M23:** Determine if factors other than unloading contribute to muscle atrophy during space flight.
- **Osteo 4**: We don't know the contribution of each risk factor on bone loss and recovery of bone strength, and which factors are the best targets for countermeasure application.
- **Osteo 5**: We need an in-flight capability to monitor bone turnover and bone mass changes during spaceflight.
- **BMed2**: We need to identify and validate measures to monitor behavioral health and performance during exploration class missions to determine acceptable thresholds for these measures.
- **BMed3**: We need to identify and quantify the key threats to and promoters of mission relevant behavioral health and performance during autonomous, long duration and/or long distance exploration missions.
- **BMed5**: We need to identify and validate measures that can be used for the selection of individuals that are highly resilient to the key behavioral health and performance threats during autonomous, long duration and/or long distance exploration missions.
- **Sleep Gap 10**: We need to identify the spaceflight environmental and mission factors that contribute to sleep decrements and circadian misalignment, and their acceptable levels of risk.

Background

Artificial gravity (AG) by centrifugation has the potential to mitigate physiological deconditioning caused by prolonged exposure to weightlessness. Although continuous rotation of a spacecraft to mimic Earth gravity is the surest AG solution, the implementation of 1G with little gravity gradient may not be necessary. Indeed, an alternative to continuous spacecraft rotation is to expose crewmembers to intermittent AG using an on-board short-radius centrifuge (Young et al. 2005). For intermittent AG, two of the most important requirements to define are the minimum duration and the frequency of centrifugation.

Previous investigations have studied the effects of intermittent AG using a short-radius centrifuge in human subjects deconditioned by 6º head-down tilt bed rest (HDBR) ranging from 4 to 21 days (Nyberg et al. 1966; Yajima et al. 1994; Lee et al. 1997; Iwasaki et al. 1998; Katayama et al. 2004; Iwase 2005; Young & Paloski 2007; Linnarsson et al. 2015). Results showed that daily exposure to 1-2 Gz at the heart for 0.5-2 hours was effective in mitigating orthostatic intolerance and maintaining exercise capacity after HDBR. Of particular interest is a

recent study that compared daily AG sessions generating +1 Gz at the heart for 30 minutes continuously (1 x 30 min) to 6 bouts of 5 minutes (6 x 5 min) separated by 5 minutes of rest (Linnarsson et al. 2015). The 6 x 5 min +Gz intervention was found to be the most effective in preserving orthostatic tolerance after HDBR, and appeared equivalent to a continuous 60-min exposure to +Gz stimulation in other studies. It was also better tolerated by the subjects.

Unfortunately, these HDBR studies were too short to evaluate the effectiveness of intermittent AG on bone and muscle structure and strength, and on sensorimotor deconditioning. Also, functional and cognitive performances, which are essential for the success of exploration-type space missions, were not tested during these studies.

Objectives

The primary objective of this research solicitation is to compare the protective effects of one single daily bout versus multiple daily bouts of AG on physiological functions that are affected by weightlessness. A secondary objective is to document the user's point of view, such as subjective rating of comfort/discomfort, perceived exhaustion, perceived benefits, and any other psychological issues associated with the intermittent AG protocols.

Research proposals are being solicited to characterize changes in physiological and psychological systems. Systems of interest include, but are not limited to musculoskeletal, sensorimotor, cardiovascular, cerebrovascular, ocular, functional performance, cognition and behavioral health.

In addition to the integrated physiological and psychological approach, proposers are also encouraged to include or collaborate with specialists in -omics research so that a relationship between -omics biomarkers and integrated physiology can be established between the deconditioning effects of bed rest and intermittent AG. NASA aims to use biomarkers and physiological monitoring to predict individual responses and then develop a personalized countermeasure program for deep space missions. This approach will also help to understand the mechanisms of deconditioning and the effects of gravitational unloading on the human body.

Proposals will be selected based on their scientific merits pursuant to NASA Human Research Program aims and strategies for closing knowledge gaps and mitigating health risks during spaceflight. Not all physiological, psychological and –omics aspects mentioned in this solicitation need to be included in each proposal. Potentially several proposals may be selected and then combined into a multidisciplinary study team.

References

Iwasaki K, Hirayanagi K, Sasaki T, Kinoue T, Ito M, Miyamoto A et al. (1998) Effects of repeated long duration +2Gz load on man's cardiovascular function. Acta Astronaut 42:175-183

Iwase S (2005) Effectiveness of centrifuge-induced artificial gravity with ergometric exercise as a countermeasure during simulated microgravity exposure in humans. Acta Astronaut

57:75-80
Katayama K, Sato K, Akima H, Ishida K, Takada H, Watanabe Y et al. (2004) Acceleration with exercise during head down bed rest preserves upright exercise responses. Aviat Space Environ Med 75:1029-1035
Lee S, Bennett B, Hargens A, Watenpaugh D, Ballard R, Murthy G et al. (1997) Upright exercise or supine lower body negative pressure exercise maintains exercise responses after bed rest. Med Sci Sports Exerc 29:892-900
Linnarsson D, Hughson RL, Fraser K, Clément G, Karlsson LL, Mulder E et al. (2015) Effects of an artificial gravity countermeasure on orthostatic tolerance, blood volumes and aerobic capacity after short-term bed rest. J Appl Physiol 118:29-35
Nyberg JW, Grimes RH, White WJ (1966) Consequence of heart-to-foot acceleration gradient for tolerance to positive acceleration (+Gz). Aerospace Med 37:665-668
Yajima K, Miyamoto A, Ito M, Maru R, Maeda T, Sanada E et al. (1994) Human cardiovascular and vestibular responses in long minutes and low +Gz loading by a short arm centrifuge. Acta Astronaut 33:239-252
Young L, Paloski W, Fuller C, Jarchow T (2005) Artificial Gravity as a Tool in Biology and Medicine. International Academy of Astronautics (IAA) Study Report. IAA Paris.
Young LR, Paloski WH (2007) Short radius intermittent centrifugation as a countermeasure to bed-rest and 0-G deconditioning: IMAG pilot study summary and recommendations for research. J Gravit Physiol 14:P31-33

Type of Research Solicited: Ground-Based

Please note that a maximum of <u>$700,000</u> ($350,000/year) for <u>two</u> years is available.

Specifically, proposals should address the following topic:

- By using bed rest and intermittent short-radius centrifugation during one or multiple sessions, characterize one or more of the physiologic responses of muscle, bone, sensorimotor, cardiovascular, cerebrovascular, ocular systems, and cognitive function. Establishment of multinational teams and inclusion of -omics biomarker approaches are encouraged but not mandatory; however, international participation will be considered during the second tier review by NASA.

A summary of the current evidence for these risks, and a reference list, is available at http://humanresearchroadmap.nasa.gov/evidence/.

For technical, programmatic, and solicitation matters:

Contact: Peter Norsk, M.D.; Human Health Countermeasures Element Scientist
Telephone: 281-244-5405
E-mail: peter.norsk@nasa.gov

B. Award Information

The selected proposal is expected to be funded as a research grant in one-year increments for activities typically lasting two years. The mechanism for funding each successful proposal will be a single grant, with funding allocations to participating investigators based on the submitted budget, available funds and programmatic review. The funding duration will depend on proposal requirements, peer review panel recommendations, and continuing progress of the activity. Proposals will be evaluated as described in Section D of this document. Proposals to continue or supplement existing grants, if selected, will result in a new grant.

Unless otherwise stated, it is anticipated that NASA awards will average $350,000 per year (total cost) and **cannot exceed $350,000** per year for a maximum duration of two years. NASA does not provide separate funding for direct and indirect costs; thus, the amount of the award requested is the total of all costs submitted in the proposed budget. It is estimated that the initial selections will be announced by June 2016 and the grant will be awarded in a reasonable timeframe thereafter.

C. Proposal and Submission Information

1. Source of Application Materials

All information needed to submit an electronic proposal in response to this announcement is contained in this NRA and in the companion document entitled "Guidebook for Proposers Responding to a NASA Research Announcement (NRA)" (hereafter referred to as the *Guidebook for Proposers*) that is located at:
http://www.hq.nasa.gov/office/procurement/nraguidebook/.

Additionally, applicants shall prepare proposals in accordance with the "Instructions for Responding to NASA Research Announcements," NASA Federal Acquisition Regulations (FAR) Supplement (NFS), Part 1852.235-72 (November 2004), hereafter referred to as the *NASA FAR Supplement*, that is located at:
http://www.hq.nasa.gov/office/procurement/regs/nfstocA.htm.

The information in this NRA **supersedes** and provides additional direction to that found in the *Guidebook for Proposers* and provides additional direction consistent with the *NASA FAR Supplement*. Proposals that do not conform to the standards outlined in this solicitation will be declared noncompliant and will be handled in accordance with the *NASA FAR Supplement*.

Proposal submission questions received will be answered and published in a Frequently Asked Questions (FAQ) document. This FAQ will be posted on the NSPIRES solicitation download site alongside this NRA, and will be updated periodically between submission release and the Step-2 proposal due date. Any supplemental information will also be posted alongside this NRA.

2. Content and Form of Proposal Submission

a. Registration in NASA Proposal Data System

This NRA requires that the proposer register key data concerning their intended submission with the NASA Solicitation and Proposal Integrated Review and Evaluation System (NSPIRES) located at http://nspires.nasaprs.com. **Potential applicants are urged to access this site well in advance of the proposal due date(s) of interest to familiarize themselves with its structure and enter the requested identifier information. It is especially important to note that every individual named on the proposal's *Cover Page* (see further below) must be registered in NSPIRES and that such individuals must perform this registration themselves. Team members will be asked to confirm their organization affiliation when added to a proposal.** No one may register a second party, even the PI of a proposal in which that person is committed to participate. This data site is secure and all information entered is strictly for NASA's use only.

Every organization that intends to submit a proposal to NASA in response to this NRA, including educational institutions, industry, nonprofit institutions, NASA Centers, the Jet Propulsion Laboratory, and other U.S. Government agencies, **must be registered in NSPIRES**, regardless of the electronic system used to submit proposals. Such registration must be performed by an organization's electronic business point-of-contact (EBPOC) in the Central Contractor Registry (CCR).

b. Electronic Submission

Proposals must be submitted electronically. Step-1 and Step-2 proposals must be submitted electronically by one of the officials at the PI's organization who is authorized to make such a submission. All team members must be registered in NSPIRES and confirm their organizational affiliation when added to a proposal before the PI organization official can submit. It is strongly recommended that the PI work closely with his/her team members and organization official to ensure the proposal is submitted by the due date and time listed in this solicitation. **Proposals submitted after the listed due dates and times will be declared noncompliant and will be handled in accordance with the NASA FAR Supplement.**

NSPIRES accepts fully electronic proposals through a combination of data-based information (e.g., the electronic *Cover Page* and its associated forms) and uploaded PDF file(s) that contain the body of the proposal. The website will provide a list of all elements that make up an electronic proposal, and the system will conduct an element check to identify any item(s) that is(are) apparently missing or incomplete. Proposers are particularly encouraged to begin their submission process early.

Requests for assistance in accessing and/or using this Website may be directed by E-mail to nspires-help@nasaprs.com or by telephone at 202-479-9376 Monday through Friday, 8:00 AM – 5:00 PM Eastern Time. Frequently Asked Questions (FAQs) may be accessed through the Proposal Online Help site at http://nspires.nasaprs.com/external/help.do. Tutorials of NSPIRES are available at http://nspires.nasaprs.com/tutorials/index.html.

3. Intent to Propose and Step-1 Proposals

Proposals solicited through this NRA will use a two-step proposal process for which the Notices of Intent (NOI) take the form of a required Step-1 proposal.

The NSPIRES system will guide proposers through submission of all required proposal information. **Please note that the Proposal Summary, Business Data, Program Specific Data, and Proposal Team are required Cover Page Elements for a Step-1 proposal.** The proposal summary should be between 100-300 words (4000 characters maximum) and understandable by the layman reader. Budgets should not be included with the Step-1 proposal. The project team is not considered binding for Step-1 and can be adjusted in an invited Step-2 proposal. **Failure to include any of the key components may result in return of your Step-1 proposal without review**.

To initiate a **mandatory** (not binding) Step-1 proposal:

- Log in using your NSPIRES user name and password.
- Click on Proposals under the NSPIRES Options.
- Click on the Create Proposal button.
- Select “Solicitation” to prepare a new proposal.
- Click the button for “Appendix G: Physiological and Behavioral Responses in Humans to Intermittent Artificial Gravity during Bed Rest” (NNJ15ZSA001N-AGBR).
- Follow the step-by-step instructions provided in NSPIRES to complete your Step-1 proposal.

Step-1 proposals submitted to NASA will include a synopsis of the intended research, with the total length of the proposal not to exceed two 8 ½ by 11 inch pages using a standard 12-point font and one-inch margins. This synopsis will be provided as a PDF proposal document upload, and must not be password protected or locked in any way. **Required elements** of the two-page, Step-1 application include:

(1) A clear description of the research product(s).
(2) The type of investigation (ground-based, analog definition, or flight definition).
(3) The specific aims of the proposal.
(4) An outline of the plan to accomplish the specific aims.

No additional documents should be uploaded with the Step-1 proposal. Budget and detailed program data should not be included with the Step-1 proposal. Project personnel are not considered binding for Step-1 and can be adjusted in an invited Step-2 proposal. References are not required for the Step-1 proposal, and if included, count towards the 5-page limit.

If your proposal is a resubmission, you should identify it as such in your Step-1 submission; you are not, however, required to address prior reviews unless invited to submit a full proposal. Please be aware that submission of a step-1 proposal to re-introduce a proposal invited during a previous review cycle to submit as a step-2 proposal, but not funded (i.e., a re-submission from a previous round of review), does not guarantee that this newly submitted step-1 proposal will necessarily be judged as responsive to the areas of focus in the current NRA.

Step-1 proposals are prepared by the PI or a designated representative of the PI. **Step-1 proposals are submitted by an official of the PI's organization after the PI has released the prepared proposal to the institution official.** It is strongly recommended that the PI work closely with his/her organization official to ensure the proposal is submitted by the due date and time listed in this solicitation. Proposals will not be accepted after the listed due dates except for as provided in the *NASA FAR Supplement 1852.235-72(g).*

All proposers, team members, and agency officials must be registered before proposal submission with NSPIRES regardless of the electronic system used to submit proposals.

Step-1 proposals shall be electronically submitted by the due date and time listed in Section G. Electronic submission of Step-1 proposals will be open during the period listed in Section G.

All submitters of Step-1 proposals are eligible to submit full Step-2 proposals. There will be no relevancy review or down-select based on the Step-1 proposal. PIs may not change the scope or specific aims listed in their Step-1 proposal without first contacting NASA.

4. Instructions for Preparation of Invited Step-2 Proposals

Step-2 proposals are due by the due date and time listed in Section G. **Step-2 proposals will be accepted from invited proposers only.** Invited Step-2 proposals must be submitted through the NSPIRES system.

The NSPIRES system will guide proposers through submission of all required proposal information. Select **prior-phase proposal** when creating an invited Step-2 proposal. Please note that the Proposal Summary, Business Data, Budget, and Proposal Team and Program Specific Questions are required Cover Page Elements for all Step-2 proposals. The proposal summary should be between 100-300 words (4000 characters maximum) and understandable by the layman reader. In addition to the Cover Page online budget forms, proposers are encouraged to provide expanded budgets as needed (i.e., subcontracts) as part of their budget justification (see number 11 below and the Guidebook for proposers). **For proposals with NASA civil servant team members only:** Proposers are required to enter the NASA civil servant team member name and fraction of full-time equivalent (FTE) involvement in the same field under the Item column in section F "Other Direct Costs" of the online budget. The funds requested should be entered as the Total Requested Funds for the NASA civil servant, including salary, fringe, materials, travel, etc. (see the FAQ posted alongside this document for additional budget instruction). This budget entry should be made for each year of NASA civil servant involvement, and is in addition to the agency identification under the team member section and the NASA civil servant FTE designation under the business data section.

To ensure proper Step-2 proposal transmission, please provide only **one** PDF attachment upload ordered as below. **For proposal sections 2 through 9 and section 16, specific instructions are given in this NRA (see section C.4.a though C.4.g). These specific instructions supersede those found in the NASA Guidebook for Proposers. Proposals that do not conform to these requirements may be declared noncompliant and declined without review.** For sections 10-

15, proposers are encouraged to reference the NASA Guidebook for Proposers; however, there are no specific submission compliance requirements for these sections (format, structure, page counts, etc.).

1. *Table of Contents*
2. *If applicable, inclusion of the Analog Study Resource Worksheet or Retrospective Data Request Study Feasibility Assessment Form (see HERO Overview posted on NSPIRES alongside this NRA).*
3. *Software Sharing Plan, if applicable (see HERO Overview posted on NSPIRES alongside this NRA).*
4. *Map to Human Research Roadmap (HRR) (see C.4.a below).*
5. *Human Subjects certifications, if applicable (see C.4.b below).*
6. *Response to prior review, if applicable (see C.4.c below).*
7. *Productivity of currently funded research, if applicable (see C.4.d below).*
8. *Scientific or Technical Project Description (see section C.4.f below).*
9. *References and Citations*
10. *Management Approach (see Guidebook for Proposers).*
11. *Personnel Biosketches (see Guidebook for Proposers).*
12. *Current and Pending Support (see Guidebook for Proposers).*
13. *Facilities and Equipment (see Guidebook for Proposers).*
14. *Budget Justification of Proposed Costs (see Guidebook for Proposers).*
15. *Letters of Collaboration or Support (see Guidebook for Proposers).*
16. *Appendices or Reprints (See C.4.g below).*

While the NSPIRES system allows for the upload of supporting documents as separate uploads, please provide the information above in only one PDF proposal document upload. It is essential that all PDF files generated and submitted meet NASA requirements. At a minimum, it is the responsibility of the proposer to:

1) ensure that all PDF files are unlocked and that edit permission is enabled – this is necessary to allow NSPIRES to concatenate submitted files into a single PDF document; and
2) ensure that all fonts are embedded in the PDF file and that only Type 1 or TrueType fonts are used. In addition, any proposer who creates files using TeX or LaTeX is required to first create a DVI file and then convert the DVI file to Postscript and then to PDF.

See http://nspires.nasaprs.com/tutorials/PDF_Guidelines.pdf for more information on creating PDF documents that are compliant with NSPIRES.

There is a recommended 10 MB size limit for proposals (Section 2.3(c) of the NASA Guidebook for Proposers). Large file sizes can impact the performance of the NSPIRES system. Most electronically submitted proposals will be less than 2 MB in size.

NSPIRES accepts electronic proposals through a combination of data-based information (e.g., the electronic Cover Page) and the uploaded PDF file that contains the proposal as outlined above. The NSPIRES proposal submission process ensures that a minimum set of required

proposal cover page fields are completed. Provision of the proposal summary and business data elements of the cover page will be necessary in order for the Authorized Organizational Representative (AOR) to submit the proposal to NASA. If either of these two proposal elements is incomplete, the "View Proposal/ Check Elements" function of NSPIRES will display red "error" flags and messages to alert the user to the information that is required but missing, and the "Submit Proposal" button will not be available. Although the PI will be able to release the proposal to the AOR, the proposal cannot be submitted by the AOR to NASA until these required fields are complete. Any additional information that is missing will be identified by yellow "warning" flags. Proposers are reminded to check the solicitation instructions to ensure compliance with all instructions, as adherence to these two element validation checks alone is insufficient to guarantee a compliant proposal. Additionally, in those cases where instruction in the NRA contradicts an NSPIRES warning, the NSPIRES yellow “warning” may be ignored. Proposers should follow the NRA instructions closely to help ensure submission of a compliant proposal.

The NSPIRES system is limited in the character sets that can be used in filling out on-line forms. Please refer to the on-line tutorials when using special characters. Alternatively, spell out special characters where possible (such as micro rather than the Greek symbol). Applicants are encouraged to preview their proposal prior to releasing the proposal to their designated Organization by clicking the “Generate” button at the bottom of the View Proposal Screen in NSPIRES. The “Generate” feature allows applicants to preview their entire proposal in a single PDF file prior to submittal, but it is not a required step in the submission process. Please contact the NSPIRES Help Desk for assistance with this feature (e-mail: nspires-help@nasaprs.com or telephone: 202-479-9376).

a) Human Research Program (HRP) Human Research Roadmap (HRR)

The investigator must examine and understand the research emphases outlined in this NRA and the risks identified in the HRP Human Research Roadmap (http://humanresearchroadmap.nasa.gov). Proposers must include a description as part of their proposal of how their research aims map to the identified IRP risks, gaps and deliverables. This description is limited to two pages and does not count towards the 20-page limit of the project description.

b) Special Matters

For proposals employing human subjects, assurance of compliance with provisions for human subjects is required.

Policies for the protection of human subjects in NASA sponsored research projects are described in NASA the NASA Policy Directive (NPD) 7100.8E “Protection of Human Research Subjects” (http://nodis3.gsfc.nasa.gov/displayDir.cfm?t=NPD&c=7100&s=8E)

NASA utilizes a just-in-time practice for approval of the use of human subjects. If the IRB certification is already approved at proposal submission, attach a copy of the certification as part of the proposal.

After award, a statement must be provided from the Applicant institution which identifies the

selected proposal by name and which certifies that the proposed work will meet all Federal and local requirements for human subjects. This includes relevant documentation of Institutional Review Board (IRB) approval. NASA will require current IRB certification prior to each year's award.

For delivery of any certifications received after the proposal due date, please contact Kevin Willison, Senior Scientist, NASA Research and Education Support Services, at kwillison@nasaprs.com.

c) Revised Proposals

Investigators submitting a proposal in response to this solicitation, and whose most recent submission that included similar specific aims to any NASA or NSBRI sponsored research announcement was not accepted, are required to submit an explanation of how the current proposal addresses criticisms from previous review cycles. This explanation shall be presented preceding the research description as part of the main proposal upload and is limited to two pages. This explanation should include changes to the current proposal as a result of review comments and, or explanation as to why prior review comments are not applicable to the current proposal.

Investigators resubmitting a proposal in response to this solicitation may only submit a proposal with similar hypothesis(es) and aims a total of three times (original submission plus two resubmissions). Significant changes must be made to the proposal hypothesis(es) and specific aims for consideration after the third attempt or the proposal will be declined without further review.

These two pages are not considered part of the 20-page project description. Proposal reviewers will be provided with the evaluations of prior submissions. Proposers must respond to prior criticisms relevant to any portion of the new proposal under consideration. Proposers who have questions concerning their response to a prior review are encouraged to contact Kevin Willison, Senior Scientist, NASA Research and Education Support Services, at kwillison@nasaprs.com.

d) Productivity of NASA- or NSBRI-Funded Research

Proposers (minimally the Principal Investigator) currently funded by or who have received funding within the last four (4) years from any NASA funding source must provide specifics to the productivity of the supported research including progress in experiments and research publications and new findings. This explanation should be presented preceding the research description as part of the main proposal upload and is limited to two pages. These two pages are not considered part of the 20-page project description. Related impacts, if any, to the proposed research plan should be highlighted in the body of the project description. **Proposers that request continued support that do not include this productivity section will be returned to the submitter without panel review and will not be considered for funding.**

e) Scientific or Technical Project Description Section (Project Description)

The length of the project description of the proposal shall not exceed 20 pages using standard (12 point) type. Text shall have one-inch margins. Referenced figures and tables must be included in the 20 pages of the project description; however figure captions can use a 10-point font. The

proposal shall contain sufficient detail to enable reviewers to make informed judgments about the overall merit of the proposed research and about the probability that the investigators will be able to accomplish their stated objectives with current resources and the resources requested. The hypotheses (if appropriate) and specific aims of the proposed research shall be clearly stated. If applicable, a statistical section with proper justification should be included in the project description. **Proposals that exceed the 20-page limit for the project description (inclusive of ALL figures and tables) will be declared noncompliant and will be handled in accordance with the NASA FAR Supplement. Cited literature and all other proposal sections are not considered part of the 20-page project description.** Reviewers are not required to consider information presented as appendices or to view and/or consider Web links in their evaluation of the proposal.

f) Facilities and Equipment

Proposers who wish to use a facility that is not under their direct control must submit with their proposal application a letter from the respective facility manager or organization manager that states the following:

1. The PI has permission to use the particular facility.
2. The PI will pay the respective organization for the use of the facility. The cost of the facility should be included in the letter as well as in the proposal budget.

g) Reprints and Appendices

Reprints and Appendices, if any, do not count toward the project description page limit, and are to be included following all other sections of the proposal (**reviewers are not required to consider information presented in proposal appendices**).

D. Proposal Evaluation Process

1. Step-1 Proposal Review

All Step-1 proposals submitted by the Step-1 submission deadline listed in Section G will be invited to submit a full Step-2 proposal. There will be no relevancy review or down-select based on the Step-1 proposal. PIs may not change the scope or specific aims listed in their Step-1 proposal without first contacting NASA.

2. Step-2 Proposal Intrinsic Scientific and Technical Merit

To be responsive to this research solicitation, proposed studies should produce research product(s) that address the research emphases stated in this solicitation, and lead to new knowledge within accepted scientific and technology standards.

All of the following criteria will be used in determining the merit score.

Significance:
Does this study address a research emphasis stated in this solicitation? Does the study test a

significant hypothesis or produce data that would enable a significant hypothesis to be generated? If the study is non-hypothesis driven, are the data produced needed to understand or reduce the risk addressed by the research emphasis? If the task will produce a software model or tool, how will it serve to better quantify or mitigate a risk? If the aims of the application are achieved, how well will the product(s) address the research emphases? If the aims of the application are achieved, how will scientific knowledge or technology advance?

Approach:
Are the conceptual framework, design, methods, and analyses adequately developed, well integrated, and appropriate to the aims of the project? Is the proposed approach likely to yield the desired results? Does the applicant acknowledge potential problem areas and consider alternative tactics? If applicable, has the applicant included a range of reasonable sample sizes for a proposed study with proper justification?

Statistical Plan:
A thorough statistical section must be included which includes a power analysis for the estimate of sample size and the comparison of males and females unless compelling evidence is provided that shows that no sex differences are expected. Does the study provide adequate justification for sample size? For example, is the choice of primary outcome relevant for the stated Aims? Are assumed effect magnitudes reasonable? Are assumed variability estimates reasonable? Are they estimated properly? Are they relevant for the proposed experimental design and data analysis methodology? What Type I and Type II errors are assumed? Is there room for a tradeoff here to accommodate sample size constraints and still provide useful information from the study? Do the investigators provide a reasonable data analysis plan? For example, is it appropriate for the proposed experimental design (e.g., repeated measures)? Does it address research hypotheses or aims? Is it robust to the sampling and other constraints associated with the research venue?

Risk Mitigation:
For a study quantifying risks to crew health or performance; does the study adequately improve the understanding of the adverse consequences, the probability of its occurrence, or the timeframe in which the risk must be addressed? For a study developing countermeasures, will the proposed countermeasure reduce a risk to crew health or performance, reduce the impact of the risk or reduce the resources required to mitigate it? For a study developing technology, will the research product reduce the risk to crew health or performance, reduce its impact or better define it and is the technology feasible within the confines of the operational environment?

Investigators:
Are the investigators appropriately trained and well suited to carry out this work? Is the work proposed appropriate to the experience level of the principal investigator and any co-investigators? Is the evidence of the investigators' productivity satisfactory?

Environment:
Does the scientific environment in which the work will be performed contribute to the probability of success? Do the proposed experiments take advantage of unique features of the scientific environment or employ useful collaborative arrangements? Is there evidence of institutional support?

3. Step-2 Proposal Review and Selection Processes

a. Compliance Matrix

All proposals must comply with the general requirements of the NRA as described in this solicitation, the *Guidebook for Proposers*, and the *NASA FAR Supplement*. Upon receipt, proposals will be reviewed for compliance with these requirements including:

1. Proposals will not be accepted after the due dates and times listed in this announcement except for as provided in the *NASA FAR Supplement*.
2. The proposal project description must be no more than 20 pages in length.
3. Submission of appropriate IRB certification for all proposals using human test Subjects in accordance with the Special Matters requirements listed in Section C.4.b.
4. Submission of an appropriate and justified budget for a funding period not exceeding that described in the NRA.
5. Investigators submitting a proposal in response to this solicitation, and whose most recent submission that included similar specific aims to any NASA or NSBRI sponsored research announcement was not accepted, are required to submit an explanation of how the current proposal addresses criticisms from previous review cycles. This explanation should be presented in a separate form of no more than two pages. Related changes to the research plan should be highlighted in the body of the project description as described in Section C.4.c.
6. A description of how the research aims map to the identified IRP risks and gaps as described in Section C.4.a.
7. A description that provides specifics to the productivity of the previously supported research including progress in experiments and research publications and new findings as described in Section C.4.d.
8. Submission of any required analog definition forms.
9. Inclusion of the Analog Study Resource Worksheet.
10. Submission of all other appropriate information as required by this NRA.

Note: At NASA's discretion, non-compliant proposals may be withdrawn from the review process and declined without further review. Compliant proposals submitted in response to this NRA will undergo an intrinsic scientific or technical merit review. In general, only those proposals most highly rated in the merit review process will undergo additional reviews for program balance and cost; however, at the HRP Chief Scientist's discretion, proposals with lower scores may also undergo additional reviews if they can be re-scoped and meet specific programmatic needs.

b. Scientific and Programmatic Reviews

The overall evaluation process for proposals submitted in response to this NRA will include a First Tier Merit Review and a Second Tier Program Alignment Review. The **First Tier Review** will be a merit peer review by a panel of scientific or technical subject matter experts. The number and diversity of experts required will be determined by the response to this NRA and by the variety of disciplines represented in the proposals relevant to the research emphases

described in this NRA. The merit review panel will assign ***a score from 0-100, or assign a Not Recommended for Further Consideration (NRFC)*** based upon the intrinsic scientific or technical merit of the proposal. The final score or NRFC designation will reflect the consensus of the peer review panel. After the merit review is complete the panel will be asked to include in their critique of each proposal any comments they may have concerning the proposal's budget. In general, proposals that are highly rated in the merit review process will undergo a second tier review for program alignment; however, at the HRP Chief Scientist's discretion, proposals with lower scores may also undergo a second tier review if they can be re-scoped and meet specific programmatic needs.

For NASA, the **Second Tier Review** will evaluate the programmatic balance, feasibility and cost of proposals. This review will be conducted by the HRP Chief Scientist, the HRP Element Scientists and HRP Element Managers. All applications will be reviewed with respect to:

- How relevant is the proposed work to the HRP Goals and Objectives? Is there clear added value of the proposed project to the HRP Integrated Research Plan? Does inclusion of the proposed work enhance the balance of the research portfolio?
- Does the proposed work clearly address a specific Gap in the HRP Integrated Research Plan? Is there unequivocal value of the project to the HRP?
- Does the plan have a high likelihood of progress and end-user adoption that will fill the HRP IRP Gap or make a major contribution to filling it?
- How does the value of the proposed work toward answering critical questions and achieving HRP Goals and Objectives compare to the cost?
- Does the proposed work incorporate team members from partner countries of NASA to foster international collaboration?

Note that neither a high merit score alone nor a high relevance score alone will obligate NASA to select any proposal. NASA retains the option to select proposals outside of the traditional fundable range if, in its judgment, their weaknesses in either merit or relevance can be resolved or mitigated and if it is in the best interests of the Government to do so.

In addition, analog definition proposals and flight definition proposals will undergo reviews for feasibility as described below.

c. Analog Definition Proposals

Only those analog definition proposals that are most highly rated in the merit review process will undergo additional reviews for analog feasibility. A panel of technical experts from NASA will evaluate the feasibility of carrying out the analog experiment and the potential for establishing teams of investigators to optimize utilization of human subjects, samples, data, and analog resources. This review will be conducted by technical experts familiar with the development and conduct of analog studies.

d. Selection

The information resulting from these two levels of review, as described above, will be used to prepare selection recommendations developed by the NASA HRP Chief Scientist and HRP

Program Manager. Selection for funding will be made by the NASA HRP Program Manager or his designee.

In order to optimize resources, NASA and NSBRI pursue the intentional formation of investigator partnerships between individual investigators whose experiments will leverage resources by addressing different facets of the same questions. NASA anticipates that such intentional teaming arrangements will result in better utilization of available resources to resolve specific critical questions. NASA and NSBRI strongly encourage investigators submitting applications in response to this NRA to consider identifying collaborations between individual investigators as part of the development of their individual proposals and to identify this pre-coordination in their management plan. Additional information can be referenced in the NASA FAR Supplement. Finally, NASA and NSBRI may integrate proposals if, in their judgments, the goals, objectives or products of the proposals are similar.

For some NASA research topics, NASA is considering utilizing individual research proposals to form a Virtual NASA Specialized Center of Research (VNSCOR) where NASA aligns a set of individual awards into an NSCOR like team project. Individual proposals may be selected to become Elements of a VNSCOR. Elements of the VNSCOR will also join a working group organized by NASA on the specific research topic. VNSCORs will be composed of four to six individual research elements, each with its own specific aims.

Where appropriate for analog definition or flight definition studies, NASA reserves the right to form teams of investigators whose experiments have compatible requirements for human subjects, specimens, operations, data, and treatment and sharing of biological samples. A selected investigator who becomes a member of a research team will be required to work with other team members to develop an integrated set of objectives that can be met within fiscal and analog or flight resource constraints. Development of this integrated approach may result in modification, transfer, addition or deletion of some objectives put forth in an individual proposal. Specifics associated with the definition period will be addressed with the investigator at the time of selection.

Additionally, proposals submitted in response to this solicitation found to have strong programmatic relevance and scientific merit that cannot be funded due to limited resources may be forwarded to partner programs or agencies for consideration.

E. Submission Dates

Solicitation Announcement Identifier: NRA NNJ15ZSA001N-AGBR

Step-1 Response Period: December 1, 2015 – January 4, 2016
Step-1 Proposals Due: January 4, 2016, 5 PM Eastern Time
Step-2 Response Period: January 5, 2016 – March 7, 2016
Step-2 Proposals Due: March 7, 2016, 5 PM Eastern Time
Estimated Step-2 Selection Announcement: June 2016

F. NASA Contacts

Additional technical information for the NASA programs is available from:

Mark J. Shelhamer, Sc.D.
Chief Scientist, Human Research Program
NASA Johnson Space Center (Mail Code SA2)
Houston, TX 77058
Telephone: 281-244-7330
Fax: 281-483-6089
E-mail: mark.j.shelhamer@nasa.gov

JSC Procurement Point of Contact:

La Toy J. Jones
Contracting Officer
NASA Johnson Space Center (Mail Code BH4)
Houston, Texas 77058
Telephone: 281-244-8023
Fax: 281-483-4066
Email: latoy.j.jones@nasa.gov

Additional information on the proposal submission process is available from:

NSPIRES
Telephone: 202-479-9376, Monday through Friday, 8 a.m. to 6 p.m. Eastern Time.
Email: nspires-help@nasaprs.com
Frequently Asked Questions: Available through the Proposal Online Help site at http://nspires.nasaprs.com/external/help.do.
Tutorials of NSPIRES: Available at http://nspires.nasaprs.com/tutorials/index.html

G. Summary of Key Information

Selection announcements are expected no earlier than June 2016, and selected awards will begin no earlier than October 1, 2016.

Number of new awards pending adequate proposals of merit	2
Maximum duration of awards	2 years
First day for submission of Step-1 proposals	December 1, 2015
Last day for submission of Step-1 proposals	January 4, 2016
First day for submission of Step-2 proposals	January 5, 2016
Last day for submission of Step-2 proposals	March 7, 2016
Page limit for the central Science-Technical section of Step-1 proposal	2 pages
Page limit for the central Science-Technical section of Step-2 proposal	20 pages
Relevance to NASA	This program is relevant to the human health and performance strategic goals and subgoals in NASA's *Strategic Plan*; see https://www.nasa.gov/sites/default/files/files/FY2014_NASA_SP_508c.pdf. Proposals that are relevant to this program are, by definition, relevant to NASA.
General information and overview of this solicitation	See Human Exploration Research Opportunities (HERO) Overview posted http://nspires.nasaprs.com
Detailed instructions for the preparation and submission of proposals	See NASA Guidebook for Proposers at http://www.hq.nasa.gov/office/procurement/nraguidebook/
Submission medium	Electronic proposal submission is required; no hardcopy is required. See also HERO Overview and Chapter 3 of the *NASA Guidebook for Proposers*.
Web site for submission of proposal via NSPIRES	http://nspires.nasaprs.com/ (help desk available at nspires-help@nasaprs.comor (202) 479-9376)
NASA point of contact concerning HRP matters	Mark J. Shelhamer, Sc.D.
NASA point of contact concerning technical	Peter Norsk, M.D.

content	
NASA point of contact concerning procurement matters	La Toy Jones
NRESS point of contact concerning submission matters	Kevin Willison
